# Usefulness of the serum creatinine/cystatin C ratio as a blood biomarker for sarcopenia components among age groups in community‐dwelling older people: The SONIC study

**DOI:** 10.1111/ggi.14876

**Published:** 2024-04-15

**Authors:** Wen Fang, Kayo Godai, Mai Kabayama, Yuya Akagi, Michiko Kido, Hiroshi Akasaka, Yoichi Takami, Kazunori Ikebe, Yasumichi Arai, Yukie Masui, Tatsuro Ishizaki, Saori Yasumoto, Yasuyuki Gondo, Koichi Yamamoto, Yasuharu Tabara, Kei Kamide

**Affiliations:** ^1^ Division of Health Sciences Osaka University, Graduate School of Medicine Osaka Japan; ^2^ Department of Geriatric and General Medicine Osaka University, Graduate School of Medicine Osaka Japan; ^3^ Department of Prosthodontics, Gerontology, and Oral Rehabilitation Osaka University Graduate School of Dentistry Osaka Japan; ^4^ Center for Supercentenarian Medical Research Keio University School of Medicine Tokyo Japan; ^5^ Research Team for Human Care Tokyo Metropolitan Institute of Gerontology Tokyo Japan; ^6^ Department of Clinical Thanatology and Geriatric Behavioral Sciences Osaka University, Graduate School of Human Sciences Osaka Japan; ^7^ Center for Genomic Medicine Kyoto University Graduate School of Medicine Kyoto Japan; ^8^ Graduate School of Public Health Shizuoka Graduate University of Public Health Shizuoka Japan

**Keywords:** creatinine/cystatin C ratio, general population, late‐stage older adults, sarcopenia, skeletal muscle mass index

## Abstract

**Aim:**

The serum creatinine/cystatin C ratio (CCR) or sarcopenia index is considered a useful marker of muscle mass. However, its usefulness in late‐stage older adults remains unclear. We aimed to determine the usefulness of CCR as an indicator of sarcopenia in community‐dwelling Japanese adults aged >75 years.

**Methods:**

Our study recruited participants aged 70, 80, and 90 ± 1 years during the baseline years, and included a 3‐year follow‐up in the Septuagenarians, Octogenarians, Nonagenarians, Investigation with Centenarians study. From 2015 to 2018, 955 participants were eligible: 367 in their 70s, 304 in their 80s, and 284 in their 90s. The diagnostic components of sarcopenia, including “low muscle mass, plus low muscle strength, and/or low physical performance,” were evaluated using the bioelectrical impedance analysis‐measured skeletal muscle mass index (SMI), handgrip strength, and short physical performance battery (SPPB) score, respectively, in accordance with the Asia Working Group for Sarcopenia 2019 criteria. Separate analyses were performed between each component and CCR, adjusting for sex, body mass index, and other blood biomarkers in each group.

**Results:**

The relationship between CCR and sarcopenia components was significant for handgrip strength (*β* = 0.21, 0.13, 0.19, and *P* < 0.0001, =0.0088, <0.0001, for the 70s, 80s, and 90s age groups, respectively); however, it was limited for SMI (*β* = 0.14; *P* = 0.0022, only for the 90s) and not significant for the SPPB score.

**Conclusion:**

CCR is a limited indicator of sarcopenia in late‐stage older adults. Although its association with muscle strength was significant, its relationship with muscle mass and physical performance was less pronounced. **Geriatr Gerontol Int 2024; 24: 529–536**.

## Graphical abstract

The creatinine/cystatin C ratio (CCR) is significantly associated with grip strength but shows limited association with muscle mass and physical performance. We recommend careful consideration when evaluating sarcopenia components using specific blood biomarkers or derived indices.

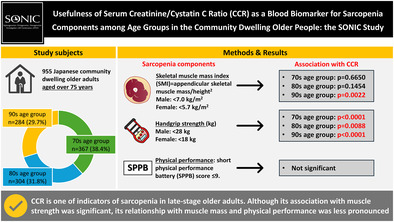

## Introduction

Sarcopenia, a common syndrome associated with aging,^1^ is prevalent not only in older people but also in critically ill patients.^2^, ^3^, ^4^, ^5^ This condition has been associated with poor long‐term health outcomes in these populations,^6^, ^7^, ^8^ highlighting the importance of its early identification.

The current international consensus states that a low muscle mass, which is typically assessed using computed tomography, magnetic resonance imaging, dual‐energy X‐ray analysis, or bioelectrical impedance analysis (BIA), is required for the diagnosis of sarcopenia.^1^, ^9^ In other words, the diagnosis of sarcopenia requires specialized medical equipment, the use of which is either unfeasible, expensive, or carries the risk of radiation. Consequently, several attempts have been made in the recent years to identify sarcopenia by focusing on the effectiveness of specific blood biomarkers or indices derived from them.^10^, ^11^, ^12^, ^13^, ^14^, ^15^, ^16^, ^17^, ^18^, ^19^, ^20^

In 2012, Tetsuka et al.^10^ suggested that the serum creatinine (Cr)/cystatin C (CysC) ratio (CCR) could be used as a readily available indicator of the residual muscle mass in amyotrophic lateral sclerosis. Subsequently, Kashani et al. validated the correlation between CCR and muscle mass in intensive care with normal renal function and defined it as the sarcopenia index in 2017.^11^ Subsequently, the value of this ratio has been verified in various situations and diseases.^12^, ^13^, ^14^, ^15^, ^16^ However, the use of CCR as an indicator of low muscle mass in community‐dwelling older adults remains controversial. Several studies in Japan have suggested that CCR is a useful marker for identifying low muscle mass in older adults.^17^, ^18^, ^19^, ^20^ In contrast, a study from China reported that CCR could not accurately detect low muscle mass in this demographic.^21^ However, the study populations in existing studies are relatively younger, averaging ~70 years of age, and there have been no studies investigating this diagnostic/relationship across different age categories within this demographic, including in extremely old individuals aged >90 years.

Given this background, we investigated the associations between CCR and sarcopenia components in community‐dwelling older Japanese people aged >75 years in the Septuagenarians, Octogenarians, Nonagenarians, Investigation with Centenarians (SONIC) study.^22^ We aimed to determine the usefulness of CCR as a marker of sarcopenia in this population.

## Methods

### Study population

The data for this study were sourced from the SONIC study, a longitudinal cohort study focused on community‐dwelling older adults in their 70s, 80s, 90s, and 100s in the eastern and western areas of Japan.^22^ The study was approved by the institutional review boards of Osaka University Graduate Schools of Medicine, Dentistry, and Human Sciences and the Tokyo Metropolitan Institute of Gerontology (approval numbers: 266, H22‐E9, 22 018, and 38, respectively).

**Figure 1:**
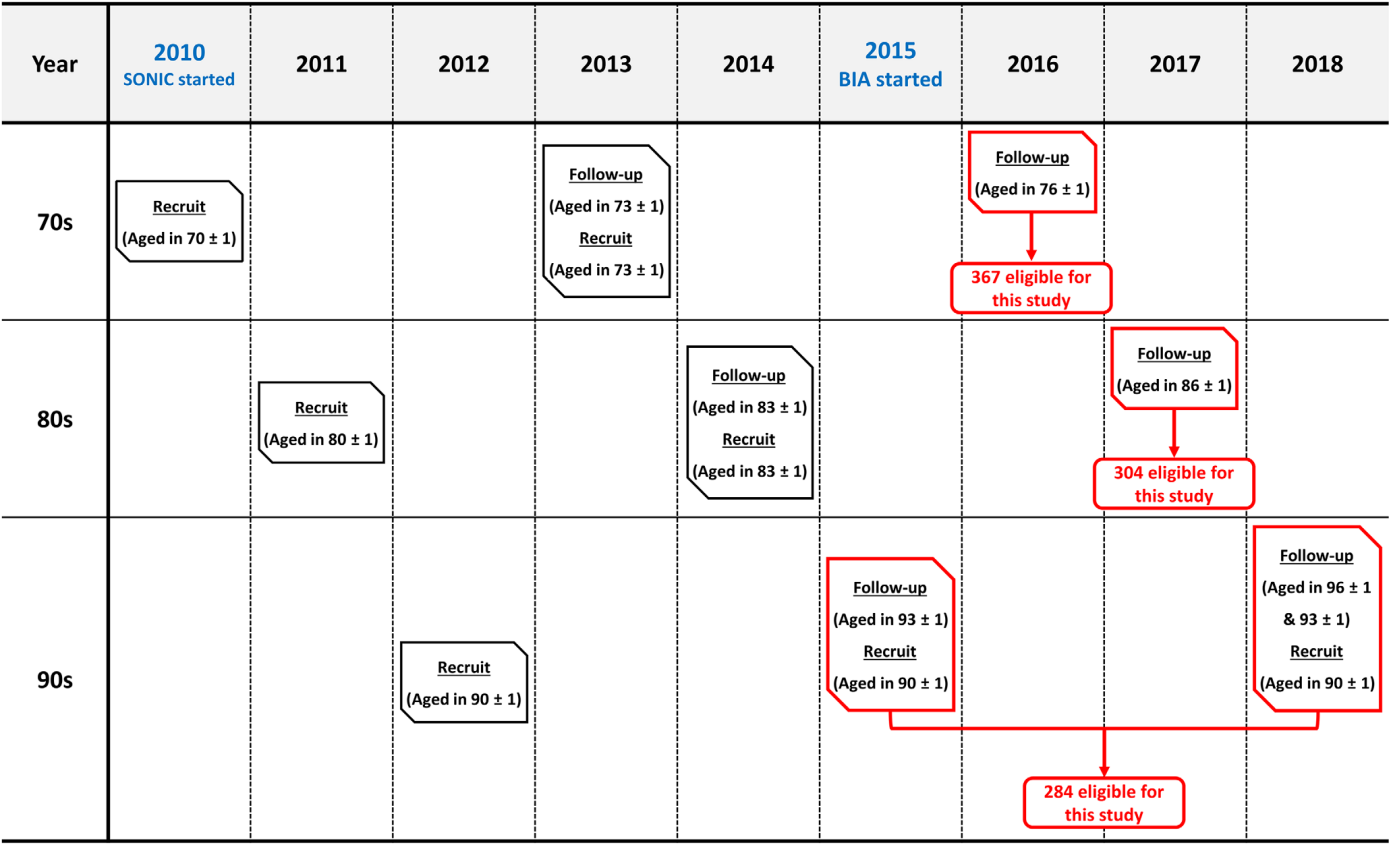
Overview of the septuagenarians, octogenarians, nonagenarians, investigation with centenarians (SONIC) study and details of participants in this study.

The SONIC study started in 2010, recruited participants aged 70, 80, and 90 ± 1 years in the baseline years, and included a 3‐year follow‐up. The baseline years for the 70s, 80s, and 90s were 2010, 2011, and 2012, respectively. To ensure an adequate number of participants in each age group, additional recruitments for the 70s and 80s age groups were conducted in 2013 and 2014. The newly recruited participants were of the same age as those in their respective first follow‐up (73 ± 1 and 83 ± 1 years old). In particular, owing to the scarcity of individuals in their 90s and the low follow‐up rate, new participants aged 90 ± 1 years were recruited at each follow‐up survey after the baseline year for the 90s age group (Fig. 1).

### Diagnosis of sarcopenia

Based on the definition by the Asia Working Group for Sarcopenia (AWGS) in 2019, the diagnostic components of sarcopenia include “low muscle mass, plus low muscle strength, and/or low physical performance.” In this study, these components were diagnosed based on a low skeletal muscle mass index (SMI), reduced handgrip strength, and a low short physical performance battery (SPPB) score. Body composition was assessed using a multifrequency BIA device (Model MC‐780A; TANITA Ltd, Tokyo, Japan). SMI was calculated by dividing the sum of the appendicular skeletal muscle mass in the four limbs (kg) by the square of the height (m^2^). Low SMI was defined as an SMI <7.0 kg/m^2^ in men and <5.7 kg/m^2^ in women. Low muscle strength was defined as a handgrip strength <28 kg for men and <18 kg for women. The criterion for low physical performance was an SPPB score ≤9.^1^

### Laboratory tests

Routine laboratory tests were performed on all participants who consented to their blood being drawn. Renal function, nutritional status, anemia, and blood glucose levels were selected as covariates. These included the serum levels of uric acid (UA), blood urea nitrogen (BUN), total cholesterol (T‐Cho), total protein (TP), albumin (Alb), hemoglobin (Hb), glucose (Glu), and hemoglobin A1c (HbA1c). In addition, to calculate the CCR according to previous reports,^10^, ^11^ we measured serum CysC levels using the gold colloid agglutination method (SRL, Inc., Tokyo, Japan).

### Assessment of renal function

The serum Cr and CysC levels were used to calculate the estimated glomerular filtration rate (eGFR). The eGFR by Cr level was calculated using the Japanese coefficient modified modification of diet in renal disease study equation:^23^ eGFRcr (mL/min/1.73 m^2^) = 194 × Cr (mg/dL)^−1.094^ × age ^−0.287^ × 0.739 (if female). The eGFR by CysC level was calculated using the Japanese coefficient modified chronic kidney disease epidemiology collaboration equation for CysC:^24^ estimated glomerular filtration rate cystatin C (eGFRcys) (mL/min/1.73 m^2^) = [104 × CysC (mg/L)^−1.019^ × 0.996^age^ × 0.929 (if female)] − 8.

### Analytical sample

We selected participants who underwent laboratory tests and had residual blood samples for additional CysC measurements. Owing to the introduction of BIA measurement in 2015, our study was limited to data collected from that time forward, and we excluded participants who did not undergo BIA measurements for any reason. In addition, because the serum Cr is produced by skeletal muscle and CysC by all nucleated cells, and both biomarkers are eliminated through the kidney, participants with poor renal function (both eGFRcr and eGFRcys <30 mL/min/1.73 m^2^,^23^, ^24^) were considered ineligible. Finally, 955 participants were enrolled in this study, comprising 367 participants selected from the follow‐up of participants in their 70s in 2016 (aged 76 ± 1 years), 304 participants in their 80s in 2017 (aged 86 ± 1 years), and 284 participants in their 90s in 2015 and 2018 (aged 91 ± 1 years). A comprehensive overview of the participants is shown in Fig. 1.

### Statistical analysis

Means and standard deviations are shown for continuous data, and numbers and percentages are shown for categorical data. For comparisons among the three age groups, the Kruskal–Wallis test was used for continuous variables, and the chi‐square test was used for categorical variables. The relationships between each sarcopenia component and sex, body mass index (BMI), CCR, and other blood biomarkers were analyzed using univariate analysis. Subsequently, multivariate regression analyses were performed to calculate the independently associated factors for each component, using variables found to be significant in the univariate analyses. All statistical analyses were performed using the JMP® 17 software package (SAS Institute Inc., Cary, NC, USA), and *P*‐values of <0.05 were considered statistically significant.

## Results

### Characteristics of study participants

The characteristics of the study participants, grouped by age, are summarized in Table 1. The sex distribution did not differ significantly between the groups. The prevalence of sarcopenia significantly increased with advancing age, culminating in the highest incidence in the 90s age group, in which more than half of the participants had sarcopenia. Additionally, both Cr and CysC levels significantly increased with age, whereas CCR decreased with age.

**Table 1:** Characteristics of the study participants according to the age groups of 70s, 80s, and 90s

	70s age group	80s age group	90s age group	
Variables	(*n* = 367, aged 76 ± 1)	(*n* = 304, aged 86 ± 1)	(*n* = 284, aged 91 ± 1)	*P*‐value
Male, *n* (%)	191 (52.0%)	163 (53.6%)	146 (51.4%)	0.8515
BMI, kg/m^2^	22.8 ± 3.1	22.2 ± 3.1	22.0 ± 3.0	**0.0095**
SMI, kg/m^2^	7.03 ± 1.11	6.62 ± 1.03	6.29 ± 1.13	**<0.0001**
Handgrip strength, kg	25.4 ± 7.9	20.9 ± 6.4	18.8 ± 7.0	**<0.0001**
SPPB score ≤ 9	49 (13.4%)	96 (32.3%)	170 (64.4%)	**<0.0001**
AWGS 2019 Sarcopenia, *n* (%)	39 (10.6%)	105 (34.5%)	154 (54.2%)	**<0.0001**
Cys C, mg/dL	0.10 ± 0.02	0.13 ± 0.04	0.13 ± 0.03	**<0.0001**
Cr, mg/dL	0.80 ± 0.20	0.88 ± 0.25	0.87 ± 0.23	**<0.0001**
CCR	7.76 ± 1.60	7.17 ± 1.45	6.98 ± 1.39	**<0.0001**
eGFRcys	66.1 ± 16.7	52.7 ± 15.9	50.2 ± 13.3	**<0.0001**
UA, mg/dL	5.33 ± 1.37	5.51 ± 1.34	5.36 ± 1.32	0.1952
BUN, mg/dL	17.7 ± 4.3	19.9 ± 5.6	20.1 ± 5.3	**<0.0001**
T‐Cho, mg/dL	200.0 ± 32.2	198.1 ± 35.5	195.5 ± 34.4	0.3122
TP, g/dL	7.26 ± 0.42	7.35 ± 0.45	7.29 ± 0.46	**0.0419**
Alb, g/dL	4.38 ± 0.31	4.18 ± 0.30	4.18 ± 0.30	**<0.0001**
Hb, g/dL	13.7 ± 1.3	13.3 ± 1.3	13.0 ± 1.4	**<0.0001**
Glu, mg/dL	116.8 ± 33.4	122.0 ± 38.1	126.4 ± 48.5	**0.0069**
HbA1c, %	5.97 ± 0.58	5.87 ± 0.60	6.06 ± 0.86	**0.0015**

Data are presented as mean ± standard deviation for continuous data and as *n* (%) for categorical data. Bold *P*‐value means significant (*P*‐value <0.05).

Alb, albumin; BMI, body mass index; BUN, blood urea nitrogen; CCR, creatinine/cystatin C ratio; Cr, creatinine; CysC, cystatin C; eGFRcys, estimated glomerular filtration rate; Glu, glucose; Hb, hemoglobin; HbA1c, hemoglobin A1c; SMI, skeletal muscle mass index; SPPB, short physical performance battery; T‐Cho, total cholesterol; TP, total protein; UA, uric acid.

### Univariate and multivariate regression analyses of sarcopenia components

The simple correlations between BIA‐measured SMI and sex, BMI, CCR, and other blood biomarkers were examined, as detailed in Model 1 (Table 2). Significant factors were used to perform further multivariate regression analyses (Models 2 and 3 in Table 2). In the 70s age group, significant correlations with SMI were observed for male sex, BMI, TP, and Hb levels. However, CCR did not show a significant correlation (*P* = 0.6650). Similarly, in the 80s age group, male sex, BMI, and Hb levels were significantly associated with SMI, whereas CCR remained nonsignificant (*P* = 0.1454). In contrast, in the 90s age group, although male sex and BMI continued to show significant correlations with SMI, Hb levels were no longer significant. CCR was a significant factor for SMI (*P* = 0.0022).

**Table 2:** Univariate and multivariate regression analysis of SMI and characteristics of study participants in the age groups of 70s, 80s, and 90s

	Model 1		Model 2		Model 3	
Variables in each age group	*r*	*P*‐value	β	*P*‐value	β	*P*‐value
70s age group						
Male	0.66	**<0.0001**	0.66	**<0.0001**	0.65	**<0.0001**
BMI, kg/m^2^	0.65	**<0.0001**	0.62	**<0.0001**	0.62	**<0.0001**
CCR	0.33	**<0.0001**	0.01	0.7078	0.01	0.6650
UA, mg/dL	0.35	**<0.0001**	−0.04	0.1359		
BUN, mg/dL	0.18	**0.0005**	0.05	**0.0498**	0.04	0.0853
T‐Cho, mg/dL	−0.31	**<0.0001**	−0.02	0.5047		
TP, g/dL	−0.16	**0.0017**	−0.12	**<0.0001**	−0.12	**<0.0001**
Alb, g/dL	−0.10	**0.0497**	0.01	0.7824		
Hb, g/dL	0.32	**<0.0001**	−0.12	**<0.0001**	−0.12	**<0.0001**
Glu, mg/dL	0.07	0.1940				
HbA1c, %	0.11	**0.0301**	−0.03	0.2320		
80s age group						
Male	0.63	**<0.0001**	0.55	**<0.0001**	0.57	**<0.0001**
BMI, kg/m^2^	0.67	**<0.0001**	0.59	**<0.0001**	0.60	**<0.0001**
CCR	0.33	**<0.0001**	0.05	0.1408	0.05	0.1454
UA, mg/dL	0.30	**<0.0001**	0.01	0.7385		
BUN, mg/dL	0.11	0.0700				
T‐Cho, mg/dL	−0.28	**<0.0001**	−0.05	0.1094		
TP, g/dL	−0.10	0.0823				
Alb, g/dL	−0.06	0.3455				
Hb, g/dL	0.15	**0.0120**	−0.10	**0.0022**	−0.11	**0.0004**
Glu, mg/dL	0.07	0.2514				
HbA1c, %	0.02	0.7375				
90s age group						
Male	0.62	**<0.0001**	0.50	**<0.0001**	0.51	**<0.0001**
BMI, kg/m^2^	0.49	**<0.0001**	0.44	**<0.0001**	0.44	**<0.0001**
CCR	0.43	**<0.0001**	0.15	**0.0019**	0.14	**0.0022**
UA, mg/dL	0.18	**0.0023**	−0.02	0.5661		
BUN, mg/dL	0.07	0.2310				
T‐Cho, mg/dL	−0.18	**0.0025**	−0.08	**0.0427**	−0.08	0.0541
TP, g/dL	−0.06	0.3211				
Alb, g/dL	0.03	0.5852				
Hb, g/dL	0.29	**<0.0001**	0.04	0.3979		
Glu, mg/dL	0.07	0.2477				
HbA1c, %	0.06	0.3326				

Model 1: Simple correlations between BIA‐measured SMI and sex, BMI, CCR, and other blood biomarkers. Model 2: Multivariate regression analysis of BIA‐measured SMI and CCR and significant factors in Model 1. Model 3: Multivariate regression analysis of BIA‐measured SMI and CCR and significant factors in Model 2. Bold *P*‐value means significant (*P*‐value <0.05).

Alb, albumin; BMI, body mass index; BUN, blood urea nitrogen; CCR, creatinine/cystatin C ratio; Glu, glucose; Hb, hemoglobin; HbA1c: hemoglobin A1c; T‐Cho, total cholesterol; TP, total protein; UA, uric acid.

We conducted identical analyses of handgrip strength (Table 3) and SPPB scores. CCR emerged as a stronger associated factor than BMI across all age groups (*P* < 0.0001, =0.0088, and <0.0001 for the 70s, 80s, and 90s age groups, respectively). Notably, in the 80s age group, BMI was not a significant factor for handgrip strength. Males were the predominant factor in all age groups. Regarding the SPPB score, no factors were identified as significantly associated with scores of 9 or less.

**Table 3:** Univariate and multivariate regression analysis of handgrip strength and characteristics of study participants in the age groups of 70s, 80s, and 90s

	Model 1		Model 2		Model 3	
Variables in each age group	*r*	*P*‐value	β	*P*‐value	β	*P*‐value
70s age group						
Male	0.73	**<0.0001**	0.58	**<0.0001**	0.61	**<0.0001**
BMI, kg/m^2^	0.15	**0.0032**	0.08	**0.0235**	0.09	**0.0082**
CCR	0.55	**<0.0001**	0.20	**<0.0001**	0.21	**<0.0001**
UA, mg/dL	0.34	**<0.0001**	0.00	0.9081		
BUN, mg/dL	0.10	0.0652				
T‐Cho, mg/dL	−0.16	**0.0026**	0.02	0.6379		
TP, g/dL	−0.05	0.3396				
Alb, g/dL	0.09	0.0753				
Hb, g/dL	0.45	**<0.0001**	0.06	0.1399		
Glu, mg/dL	0.02	0.6890				
HbA1c, %	0.02	0.7477				
80s age group						
Male	0.68	**<0.0001**	0.62	**<0.0001**	0.62	**<0.0001**
BMI, kg/m^2^	0.10	0.0789				
CCR	0.42	**<0.0001**	0.12	**0.0240**	0.13	**0.0088**
UA, mg/dL	0.17	**0.0032**	−0.07	0.1481		
BUN, mg/dL	0.05	0.3724				
T‐Cho, mg/dL	−0.10	0.0914				
TP, g/dL	−0.01	0.8836				
Alb, g/dL	0.06	0.2823				
Hb, g/dL	0.29	**<0.0001**	0.08	0.1111		
Glu, mg/dL	0.07	0.2034				
HbA1c, %	0.00	0.9717				
90s age group						
Male	0.74	**<0.0001**	0.60	**<0.0001**	0.63	**<0.0001**
BMI, kg/m^2^	0.18	**0.0029**	0.12	**0.0019**	0.13	**0.0008**
CCR	0.56	**<0.0001**	0.20	**<0.0001**	0.19	**<0.0001**
UA, mg/dL	0.20	**0.0006**	−0.03	0.4737		
BUN, mg/dL	0.05	0.3844				
T‐Cho, mg/dL	−0.09	0.1559				
TP, g/dL	0.01	0.8797				
Alb, g/dL	0.17	**0.0044**	0.12	**0.0029**	0.15	**0.0002**
Hb, g/dL	0.39	**<0.0001**	0.08	0.0667		
Glu, mg/dL	0.09	0.1269				
HbA1c, %	0.02	0.7136				

Model 1: Simple correlations between handgrip strength and sex, BMI, CCR, and other blood biomarkers. Model 2: Multivariate regression analysis of handgrip strength, CCR, and the significant factors in Model 1. Model 3: Multivariate regression analysis of handgrip strength, CCR, and the significant factors in Model 2. Bold *P*‐value means significant (*P*‐value <0.05).

Alb, albumin; BMI, body mass index; BUN, blood urea nitrogen; CCR, creatinine/cystatin C ratio; Glu, glucose; Hb, hemoglobin; HbA1c: hemoglobin A1c; T‐Cho, total cholesterol; TP, total protein; UA, uric acid.

## Discussion

In this study, we demonstrated that the usefulness of CCR as a marker of sarcopenia in late‐stage older adults is limited. Although CCR was significantly associated with handgrip strength across all age categories, its correlation with SMI was observed only in the 90s age group. Furthermore, there was no significant relationship between the CCR and SPPB scores in any age group.

The prevalence of sarcopenia varies among studies, depending on the definitions used, and few studies have reported its prevalence in late‐stage older people.^25^ A recent study from Japan revealed that ~22% of both men and women aged 75–79 years old and 32.4% of men and 47.7% of women aged ≥80 years are affected by sarcopenia.^26^ In this study, the participants were community‐dwelling older adults who were able to complete their study visits, indicating relatively good health and physical performance. The prevalences of sarcopenia in the 70s and 80s age groups were lower than those reported in previous studies (10.6% and 34.5%, respectively). However, sarcopenia was prevalent in 54.2% of participants in their 90s. We speculate that adjusting the diagnostic criteria for sarcopenia according to age category in late‐stage older adults could provide a more accurate representation of their physical function.

Our results indicate that male sex is one of the predominant factors correlated with SMI, a finding consistent with those of previous studies.^17^, ^18^, ^19^, ^20^ Another observation regarding sex was that the correlation coefficient between male sex and SMI decreased with age. In general, skeletal muscle mass tends to be greater in males than in females. Changes in skeletal muscle mass have a lower impact on serum CysC levels than on Cr levels. Therefore, changes in CCR due to reduced skeletal muscle mass may be greater in males than in females. In contrast, skeletal muscle mass declines with age,^27^ with a greater decrease observed in older men than in older women.^28^ Consequently, as age increases, the difference in skeletal muscle mass between sexes may diminish, potentially leading to a weakened correlation with SMI.

The correlation coefficients between BMI and SMI are quite high, given that SMI is dependent on body weight.^17^ Similar to the correlation with sex, the correlation coefficient between BMI and SMI decreased with age. This trend could be attributed to the fact that muscle mass typically peaks in the fourth decade and subsequently declines, leading to more weight being gained as fat rather than as lean mass.^27^ However, this characteristic of weight gain in older adults could lead to fat infiltration in the muscle, a condition known as myosteatosis, which has been found to be associated with muscle strength and all‐cause mortality.^29^ The mean BMI of each age group was ~22 kg/m^2^, which is consistent with the ideal BMI for the Japanese population.^30^ Furthermore, according to the Dietary Reference Intakes for Japanese (2020) by the Japan Ministry of Health, Labor, and Welfare, the ideal BMI range for Japanese individuals aged >65 years is 21.5–24.9 kg/m^2^.^31^ Based on our results, compared with the impact of BMI on muscle mass, its influence on handgrip strength was markedly diminished, especially in the 80s age group, where no significant correlation was observed. Therefore, for older individuals, maintaining a healthy weight is likely not synonymous with sufficient muscle strength. Recently, the AWGS introduced the term “possible sarcopenia,” characterized by low muscle strength, or reduced physical performance, but not accompanied by low muscle mass.^1^ For affected individuals, the AWGS recommends lifestyle changes, including diet modification, exercise, and health education, because muscle strength is more important than muscle mass in estimating the mortality risk.^29^ Our results emphasize the importance of evaluating “possible sarcopenia” for late‐stage older adults.

Recently, the correlation between CCR, also known as the sarcopenia index, and skeletal muscle mass has been verified in numerous patients and community‐dwelling older adults.^10^, ^11^, ^12^, ^13^, ^14^, ^15^, ^16^, ^17^, ^18^, ^19^, ^20^ However, based on our results, the correlation between CCR and SMI was significant only in the 90s age group, in which the prevalence of sarcopenia was highest and the SMI was lowest. Evaluating skeletal muscle mass by calculating the SMI using BIA measurements requires a multifrequency device, which is not commonly available in clinical settings. Moreover, older adults often face difficulties in visiting medical institutes owing to reduced physical function. Although most participants in our study could complete their study visits independently, a considerable number of participants, especially those above 90 years, required accompaniment by others. Therefore, the older an individual is, the more beneficial it becomes to detect the presence of sarcopenia using only a blood test. Our results suggest the potential usefulness of CCR in evaluating muscle mass among extremely old individuals aged over 90 years. However, BIA measurements require a calibration equation, because BIA does not directly measure specific body components. The software equation in current BIA devices in Japan was derived from a cohort of 756 Japanese individuals aged 18–86 years.^32^ Therefore, there could be potential inaccuracies when it is applied to individuals aged over 90 years. Thus, our findings regarding those above 90 years should be approached with caution and will require validation in future studies. Additionally, there is a pressing need to develop a BIA calibration equation specifically for populations older than 90 years.

The association between CCR and handgrip strength was significant across all age categories, which is consistent with the results of previous studies.^19^, ^20^ Moreover, the correlation coefficients in our study were even higher than those for BMI, which is inconsistent with earlier findings. This discrepancy may stem from the age differences between our study and other studies. Our findings demonstrate that CCR serves as a reliable indicator for assessing muscle strength in late‐stage older adults, and its association with muscle strength is stronger than that of BMI in this demographic. Some inflammatory markers, such as C‐reactive protein, interleukin‐6, and tumor necrosis factor α, have been reported to be associated with lower skeletal muscle strength.^33^, ^34^ Thus, a novel muscle strength evaluation index that combines CCR with other inflammatory markers should be developed through further studies.

Other blood biomarkers worth noting, which are significantly associated with SMI or handgrip strength, include serum Alb and Hb levels. Their associations were inconsistent across different age categories. The relationship between handgrip strength and serum Alb was significant only in the 90s age group, suggesting that good nutritional status becomes increasingly important for maintaining muscle strength. A similar finding was reported in a longitudinal study of 1320 community‐dwelling older adults aged 65–88 years.^35^ Our results support that perspective and provide evidence even for individuals aged >90 years. The serum Hb level showed significant relationships with SMI in both the 70s and 80s age groups, which may suggest its potential usefulness for muscle mass evaluation in late‐stage older adults. Similar findings were also reported by previous studies;^36^, ^37^, ^38^ however, we believe that further studies are needed, especially for extremely old individuals aged >90 years.

This study has several limitations. First, blood samples were collected irrespective of fasting status; consequently, the levels of certain biomarkers might have been affected. Second, reliable data regarding participant comorbidities that may be associated with sarcopenia were lacking. Therefore, specific blood indicators were introduced to mitigate this limitation. Third, the calibration equation of existing BIA devices has not been validated for individuals aged over 90 years.^32^

In conclusion, CCR has limited utility as an indicator of sarcopenia in late‐stage older adults. Although our study demonstrated its value in assessing muscle strength, its usefulness in evaluating muscle mass and physical performance was limited in this population. The usefulness of CCR identified in previous studies cannot be generalized to this demographic group. Therefore, the impact of aging should be carefully considered when evaluating sarcopenia components using specific blood biomarkers or derived indices.

## Disclosure statement

KK: Research funding (grant numbers: dk0110040, 19K11138, 19K07888, and FY2023 CiDER). YT: Research funding (grant number: dk0110040). Other authors declare no conflicts of interest.

## Author contributions

KK, YG, KI, and TI were responsible for designing the SONIC study. All authors were involved in data collection. WF performed the statistical analysis and drafted the initial manuscript. KK, KG, YT, and HA contributed significantly to manuscript revision, offering valuable comments and insights, and played a key role in data collection, interpretation, and analysis. All authors reviewed and approved the final manuscript.

## Data Availability

The data that support the findings of this study are available on request from the corresponding author. The data are not publicly available due to privacy or ethical restrictions.
